# Photoacoustic Spectroscopy Combined with Integrated Learning to Identify Soybean Oil with Different Frying Durations

**DOI:** 10.3390/s23094247

**Published:** 2023-04-25

**Authors:** Hui Luo, Kaiyun Yang, Lili Ji, Lingqi Kong, Wei Lu

**Affiliations:** College of Artificial Intelligence, Nanjing Agricultural University, Nanjing 210031, China; lh821005@njau.edu.cn (H.L.);

**Keywords:** photoacoustic spectroscopy, soybean oil, ensemble learning, free fatty acids, non-destructive testing

## Abstract

Soybean oil produces harmful substances after long durations of frying. A rapid and nondestructive identification approach for soybean oil was proposed based on photoacoustic spectroscopy and stacking integrated learning. Firstly, a self-designed photoacoustic spectrometer was built for spectral data collection of soybean oil with various frying times. At the same time, the actual free fatty acid content and acid value in soybean oil were measured by the traditional titration experiment, which were the basis for soybean oil quality detection. Next, to eliminate the influence of noise, the spectrum from 1150 cm^−1^ to 3450 cm^−1^ was selected to remove noise by ensemble empirical mode decomposition. Then three dimensionality reduction methods of principal component analysis, successive projection algorithm, and competitive adaptive reweighting algorithm were used to reduce the dimension of spectral information to extract the characteristic wavelength. Finally, an integrated model with three weak classifications was used for soybean oil detection by stacking integrated learning. The results showed that three obvious absorption peaks existed at 1747 cm^−1^, 2858 cm^−1^, and 2927 cm^−1^ for soluble sugars and unsaturated oils, and the model based on stacking integrated learning could improve the classification accuracy from 0.9499 to 0.9846. The results prove that photoacoustic spectroscopy has a good detection ability for edible oil quality detection.

## 1. Introduction

With the promotion of fast-food culture, an increasing number of people like to eat fried foods. Fry oil is used for long durations, which can lead to chemical reactions such as hydrolysis, oxidation, polymerization, and cracking, resulting in the production of polymers, peroxides, and harmful products such as aromatic hydrocarbons and free fatty acids, as well as causing nutritional loss and deterioration in quality, and the content of harmful substances increases with the duration of frying [1]. Animal experiments have shown that long-term consumption of overstandard frying oils can cause symptoms such as hepatomegaly and reduced immune function, leading to an increased risk of cardiovascular disease [2]. Therefore, it is important to study the changes in the physicochemical index of edible oil during the process of frying to ensure the quality control of frying oil and food safety.

Edible oils are rich in fatty acids, which provide essential energy to the human body, and fatty acids are divided into saturated fatty acids and unsaturated fatty acids according to their chemical structure. Existing studies on the detection of unsaturated fatty acid content in food are still dominated by manual detection and chemical analysis. Savych [3] determined the content of fatty acids and unsaturated fatty acids in specific plants by GS–MS analysis and applied the method to analyze pharmaceuticals for their anti-cholesterol, anti-inflammatory, immunomodulatory, and neuroprotective activities. However, the chemicals involved are relatively expensive, technically challenging, and time-consuming to test. This makes them unsuitable for large-scale food testing. Zhang [4] developed a GC-FID assay using a DB-FFAP capillary GC column to separate 15 fatty acids with an accuracy of 99.6%. Compared to traditional GC methods, the GC-FID method does not require derivatization. However, its robustness is still low and cannot meet current detection requirements. Akkaya [5] and others used NIR reflectance spectroscopy combined with a modified partial least squares (MPLS) and partial least squares (PLS) regression method to develop calibration equations for the determination of fatty acid composition and content in sunflower seeds. Near-infrared spectroscopy offers fast detection speed, but its sensitivity is poor. Generally, the content required for detection must be greater than 0.1%, which significantly limits the substances that can be detected.

Photoacoustic spectroscopy (PAS) [6] is a novel technology based on the principle of photothermal measurement. Different from the traditional measurement principle of absorption spectrum, PAS does not directly detect the spectrum based on the characteristics of photons. Instead, it detects the periodic heat flow generated in the non-radiative excitation phenomenon after the sample is exposed to light. At different spectral wavelengths and modulation frequencies, the thermal diffusion lengths in various materials differ. Therefore, material composition analysis can be conducted by examining light absorption in the thermal diffusion layer near the surface of the material.

Photoacoustic spectroscopy mitigates the influence of tissue scattering characteristics on the measurement results through the multidimensional combination of spectrum and acoustics, and the spectral data can be extracted without damage to the sample, which has the advantages of high speed and low cost. In recent years, PAS has been widely used in agriculture and food. Gustavo Larios [7] used Fourier transform infrared spectroscopy to obtain the content characteristics of proteins, fatty acids, and amides in soybean seeds, and he constructed the seed vigor discrimination model on this basis. The cross-validation test revealed that high-vigor soybean seeds and low-vigor seeds were successfully distinguished. Using coupled quantum cascade laser PAS, Linhares [8] identified and quantified N_2_O GHGS in the PPMV range and obtained N_2_O emission concentrations for the four mixtures. Combining PAS and chlorophyll fluorescence studies, Pontes [9] conducted metabolic characterization and photosynthetic physiological analysis using FTIR-PAS and ChlF analysis to evaluate the interaction of nanoceria when the concentration of bicarbonate ions increased. This revealed the metabolic induction reaction of CeO_2_ NPs combined with bicarbonate ions, providing new ideas for the study of photosynthetic interaction information. Zhe Xing [10] explored differences in the composition and structure of soil organic matter by infrared PCA on the micron scale as a function of probe depth.

As a group of unsaturated fatty acids in cooking oil, the level of free fatty acids is a relatively direct indicator of whether the oil is still edible [11]. Therefore, to reduce the waste of cooking oil and to avoid the risk of high levels of free fatty acids in humans, it is important to develop a method for the classification of soybean oil with different frying durations based on whether the free fatty acid content exceeds the limit. In this paper, the photoacoustic spectrum combined with the stacking integrated learning model was proposed to analyze the photoacoustic spectrum of soybean oil at different frying durations. According to the photoacoustic spectra of soybean oil fried for different durations, whether the free fatty acids of fried soybean oil exceeded the standard was determined and used as the index of soybean oil classification.

## 2. Experimental Objects and Methods

### 2.1. Materials

The frying experiments were carried out at 160 °C. The frying periods were 0 h, 8 h, 16 h, 24 h, 32 h, 40 h, 48 h, 56 h, 64 h, 72 h, and 80 h. For each frying period, 20 groups of soybean oil were randomly selected for testing. A total of 220 sets of samples were selected and sealed in test tubes with no visible residue after frying and stored in the laboratory refrigerator (4 °C) for subsequent PAS detection experiments.

### 2.2. Experimental Methods

#### 2.2.1. Frying Experiment

First, 10 L of soybean oil was poured into a frying pan, and when the oil temperature reached 160 °C, beef meatballs were added for frying. The experiment was carried out for a total of 80 h, with 10 meatballs added at 15 min intervals, the dregs filtered out at 1 h intervals, and fresh oil added to 10 L. One hundred milliliters of soybean oil was sampled at 8 h intervals during the frying period, and the percentage of total polar components in soybean oil was measured until the value exceeded 20% of the waste indicator for frying oil as specified in the local government cooking oil standard GB7102.1-2003.

#### 2.2.2. Free Fatty Acid Content Testing

The traditional method is a titration test to measure the free fatty acid content and acid value. The acid value was calculated as follows:

(1) $$X_{AV}=\frac{\left(V-V_{0}\right)\times c\times 56.1}{m}$$

where, V is the volume of the standard titration solution consumed for the sample determination; $V_{0}$ is the volume of the standard titration solution consumed for the corresponding blank determination; c is the molar concentration of the standard titration solution; 56.1 is the molar mass of potassium hydroxide; and m is the weighing volume of the oil sample.

### 2.3. PAS System

**Figure 1:**
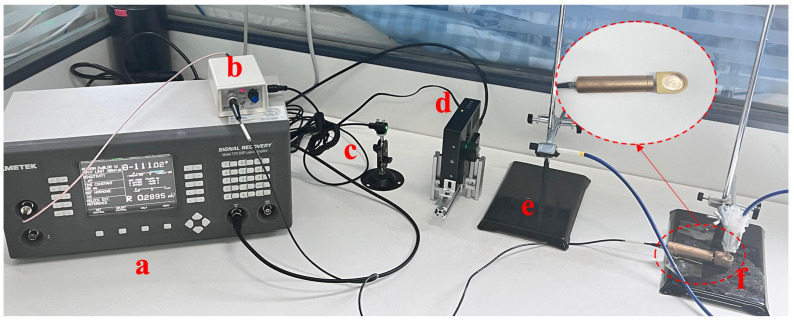
Physical picture of photoacoustic spectroscopic detection systems. a. Lock-in amplifier; b. Microphone power supply; c. Polaris infrared light source; d. Chopper; e. Optical fiber; f. Microphone and photoacoustic cell.

The PAS system used in this test was built in-house and is shown in Figure 1. The chopper type 197 and lock-in amplifier type 7270 from Amtetk were combined with a resonant photoacoustic cell developed in the laboratory to ensure that the photoacoustic signal was maximized at the receiver end of the microphone. Optical and acoustic spectroscopy of the oil samples was performed using a Polaris infrared light source and an MA221 microphone. The photoacoustic signal was collected by Omnic software. The range of the photoacoustic spectrometer was from 400 to 4000 cm^−1^, and the accuracy of the full spectral wavenumber was better than 0.005 cm^−1^.

**Figure 2:**
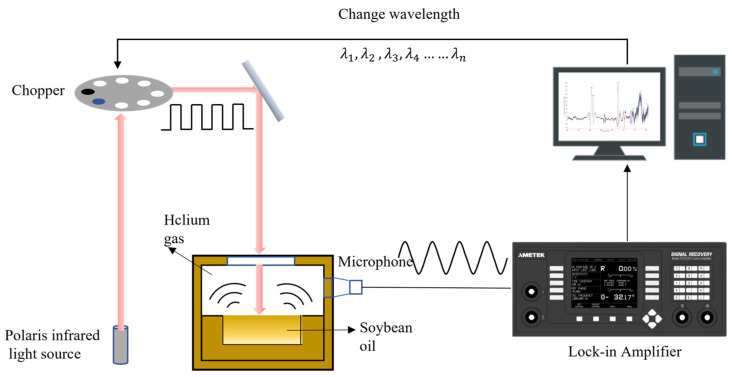
Photoacoustic spectrum detection process.

The experimental operation process is shown in Figure 2. First, before collecting the photoacoustic signal from the oil samples, the PAS system was prewarmed for 30 min to reduce the interference of water vapor to ensure the accuracy and validity of the test data. Subsequently, deep-fried soybean oil samples were placed in the photoacoustic cell, and the cell was continuously purged with helium gas for 10 s (10 mL/min) to reduce the interference of external factors such as moisture, to maintain the stability of the test process, and to reduce air humidity. The scanning resolution was set at 8 cm^−1^, and the kinetic mirror rate was set at 0.32 cm/s, with a carbon black background as the control. Finally, under the irradiation of infrared modulated light, the oil sample in the photoacoustic cell absorbed light energy and converted it into heat energy.

The independently designed resonant photoacoustic cell generates pressure wave nodes when it operates at the resonant frequency. Under the illumination of the Polaris infrared light source, the sample in the photoacoustic cell absorbs light energy and converts it into heat energy. The internal energy of the object increases, generating thermal expansion, and the heat is transmitted to the surrounding gas in the sample cell. The T-type photoacoustic cell is equipped with a resonator in addition to the absorption cell for laser intake, and a microphone is placed in the resonator to receive the photoacoustic signal. Based on this, we designed an additional resonator for the curved photoacoustic cell to ensure that the sound pressure in the photoacoustic cell is nearly zero without resonance at the resonant frequency near the pipe length of 2 cm, and the sound pressure in the microphone receiving area reaches the maximum value.

### 2.4. Spectral Data Preprocessing

In order to reduce the influence of the instrument itself and the detection environment (room noise, temperature, and humidity) on the results, this paper first applied EEMD (ensemble empirical mode decomposition) [12] to the raw spectral data, which effectively suppresses modal aliasing by superimposing Gaussian white noise for several durations in an empirical mode decomposition.

To reduce the overfitting phenomenon caused by the nonlinear superposition of spectral data due to environmental factors and the high latitude, overlap, and redundancy features of the photoacoustic spectral signal itself and to improve the speed and accuracy of computing, three commonly used dimensionality reduction algorithms, principal component analysis (PCA) [13], successive projection algorithm (SPA) [14], and competitive adaptive reweighting algorithm (CARS) [15], were used in this paper to extract the feature wavelength. PCA replaces a large number of original interrelated variables with a smaller number of uncorrelated principal components while retaining the correlation information present in the original variable data set. SPA is a forward variable selection algorithm that minimizes covariance in the vector space, using a simple projection operation in the vector space to obtain a subset of variables with small covariance. The CARS subset of wavelengths in the partial least squares regression model with large absolute values of the regression coefficients is selected, and the subset with the lowest root mean square error of cross-validation is chosen using cross-validation.

### 2.5. Modeling of Ensemble Learning Based on Stacking

**Figure 3:**
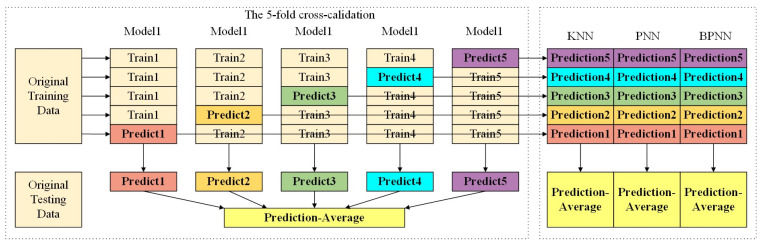
Training and prediction models for meta-learning.

To improve the accuracy of the classification model, the model was first trained and tested by feeding the processed photoacoustic spectral data into a variety of machine learning models. In addition to optimizing the parameters during the training process to improve model accuracy, the machine learning process also requires extensive cross-validation for tuning parameters prior to training. For duration series prediction, traditional cross-validation methods may result in using future data to predict current data. To avoid this data backcasting problem, this experiment was conducted in the following way, as shown in Figure 3. First, the 5-fold cross-validation was combined with the day-forward algorithm to train the later data in a stepwise manner with the previous data for validation. Second, three classifiers with higher accuracy that were closer to each other were selected from multiple weak classifiers by meta-learning as subsequent stacking integrated learning meta-models.

Stacking integration learning refers to the training of a model to combine various models to improve the accuracy of the model. To improve the generalizability of the model and to avoid the problems of model failure and poor accuracy of principal component extraction methods, this paper uses stacking integration learning to serially stack multiple weak classifiers with similar accuracy. These are extracted by cross-validation methods and weights are assigned to the different weak classifiers through a voting mechanism before training the base layer model on a complete training data set. The base layer model was trained on the full training data set and the features were then output. The features of the first layer were then combined and averaged to form a new dataset, which was used as input to the second layer of the training model, resulting in a classifier with better classification accuracy.

**Figure 4:**
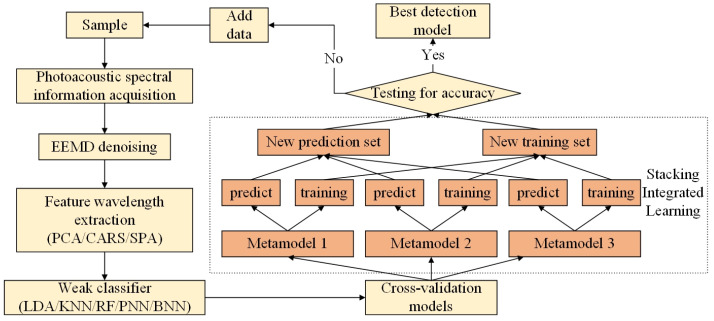
General flow chart of the grading model.

Figure 4 shows the fusion process of PCA pretreatment and the stacking integrated learning model for fried soybean oil in this experiment. First, the obtained spectral data were denoised, and then the feature wavelengths were extracted by three feature extraction methods. The obtained characteristic wavelength was used as the input of the meta-model, and the three medium-weak classifiers with high accuracy and similarity were selected. The meta-models selected in the experiment were the K-nearest neighbor (KNN) [16], product-based neural network (PNN) [17], and backpropagation neural network (BPNN) [18]. Second, the stacking integrated learning model was established. The stacking integrated learning model improved the accuracy of the model through a two-layer training model. The preprocessed spectral data set was divided into a training set and a test set. According to the ratio of 4:1, the first-layer training model was input to obtain three groups of new training sets and new test sets. The training set was the sum of the prediction results obtained by each weak classifier, and the test set was the mean of the prediction results of the three weak classifiers. The new data set was input to the second layer model. The second layer model was the weak classifier with the best modeling effect. Finally, the nondestructive testing method of soybean oil frying duration classification was obtained by using the model.

## 3. Results and Discussion

### 3.1. Results of Free Fatty Acid Content Measurements

The titration test was based on GB 5009.229–2016, which is a government-issued standard for determining whether a substance in an oil exceeds legal limits. The acid value and free fatty acid content of soybean oil with different frying durations obtained by the titration test are shown in Table 1, where the acid value is expressed as milligrams of potassium hydroxide required to neutralize free fatty acids in 1 g of oil. By comparing Table 1 data with GB7102.1-2003, it can be seen that the acid value and free fatty acid content of the soybean oil samples exceed the standard values of 1.3 mg/g and 0.6% after 48 h of frying, and 48 h is the threshold for soybean oil use. In the establishment of the stacking integrated learning model, whether the soybean frying duration reaches the threshold duration was used as the class label basis for the photoacoustic spectral classification of fried soybean oil. If the duration was greater than the threshold duration, the classification label of the spectral data was 1; otherwise, the classification was 0.

### 3.2. Spectral Information Preprocessing and Analysis

**Figure 5:**
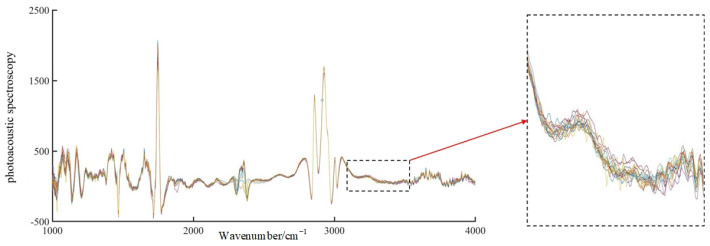
Original photoacoustic spectroscopic images of fried oil.

**Figure 6:**
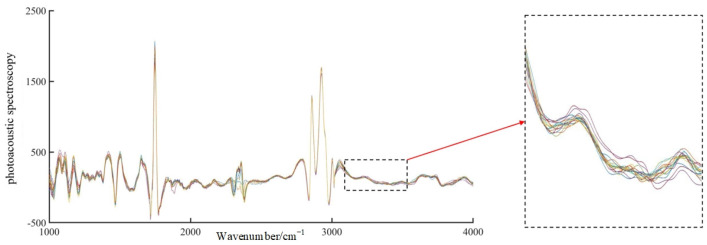
De−noised photoacoustic spectral image of fried oil.

A total of 220 sets of spectral data were collected. Figure 5 shows the raw, unprocessed spectral images of the frying durations over 48 h. As shown in Figure 5, although the spectra were collected in the wavenumber range of 400 cm^−1^ to 4000 cm^−1^, they were not analyzed due to the low signal-to-noise ratio in the wavenumber ranges from 400 cm^−1^ to 1150 cm^−1^ and from 3450 cm^−1^ to 4000 cm^−1^. The bands from 400 cm^−1^ to 1150 cm^−1^ and from 3450 cm^−1^ to 4000 cm^−1^ were not analyzed because of the low signal-to-noise ratio and the low amount of valid information. For the same durations, to reduce the interference of uncertainties, the spectral images in the wavenumber band of 1150 cm^−1^ to 3450 cm^−1^ were denoised by EEMD, and the denoised images are shown in Figure 6. The EEMD method can effectively remove the external interference, making the images smoother and the spectral features more obvious.

As seen from Figure 5, although the spectra are collected in wavenumber segments from 400 to 4000 cm, they are not analyzed due to the low signal-to-noise ratio in wavenumber segments from 400 to 1150 cm^−1^ and from 3450 to 4000 cm^−1^ with less effective information.

Figure 5 and Figure 6 show that soybean oil with different frying durations have similar absorption characteristics between 1150 and 3450 cm^−1^, and the general fluctuation trend is largely similar. There are four distinct absorption peaks between 1100 cm^−1^ and 1200 cm^−1^, 1730 cm^−1^ and 1750 cm^−1^, 2800 cm^−1^ and 2900 cm^−1^, and 2900 cm^−1^ and 3000 cm^−1^, with sharp peaks at 1747 cm^−1^, 2858 cm^−1^, and 2927 cm^−1^. All of these absorption peaks reflect the photoacoustic spectral properties of the internal components of the frying oil.

Free fatty acids are organic acids that contain chemical bonds such as C-C, C-H, O-H, and hydroxyl groups. Soybean oil contains proteins, fats, and other components in addition to the chemical reactions that occur when the free fatty acid content increases after frying. The small absorption peak in the 1700 cm^−1^ band of the characteristic spectrum is mainly caused by the superposition of the C-C bond in unsaturated oils, the stretching vibration of the C-O bond in proteins (amide I), and the bending vibration of the O-H bond in water [19]. The absorption peak at approximately 2850 cm^−1^ to 2890 cm^−1^ is the result of the superposition of the N-N bond in proteins and the O-H bond in water [20]. The C-H bond in malondialdehyde, oil, and cellulose in soybean oil results in a sharp absorption peak at 2900 cm^−1^ to 2930 cm^−1^ [20]. Therefore, the nondestructive testing model of frying oil can suitably reflect the biochemical parameters inside the oil by extracting the characteristic wavelengths.

### 3.3. Wavelength Extraction of Photoacoustic Spectral Data

#### 3.3.1. PCA Feature Wavelength Extraction

**Figure 7:**
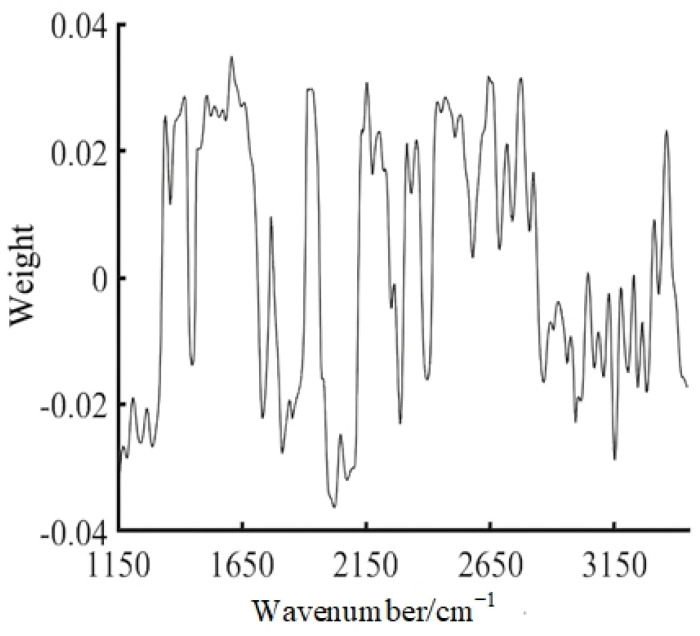
Soybean oil characteristic spectrum at 500 Hz.

**Figure 8:**
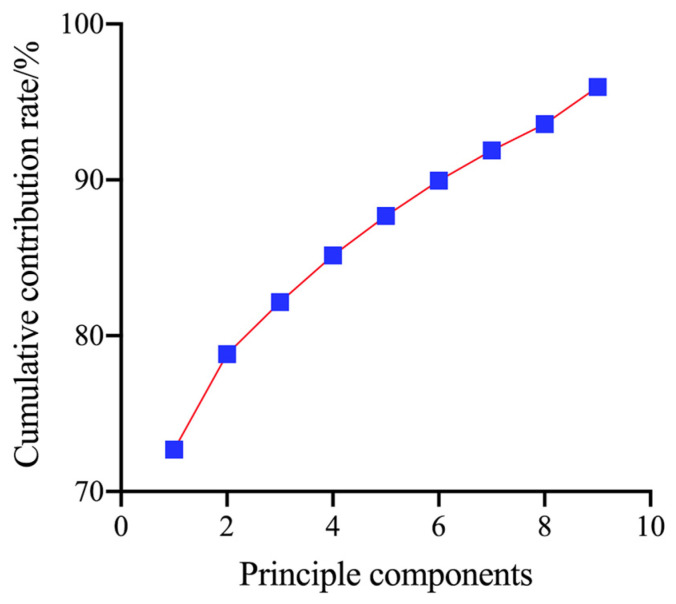
Cumulative contribution of the variance of the principal components.

To obtain a prediction model with a relatively simple input and rapid operation, this novel technique reduces the dimensionality of the data through PCA before performing the model input. The PCA method maps high−dimensional data into low−dimensional data utilizing linear projection while setting 95% as the target cumulative contribution to ensure that the characteristics of most data points are retained. Figure 7 and Figure 8 show that the cumulative contribution of the first nine components of the oil is 95.953%, which contains most of the characteristics of the oil; thus, the first nine principal components were selected instead of all the information in the spectrum to improve the accuracy of the operation. The wavenumbers of these principle components are 1427, 1469, 1558, 1693, 1916, 2183, 2476, 2530, and 2769 cm^−1^.

#### 3.3.2. CARS-Based Feature Wavenumber Extraction

**Figure 9:**
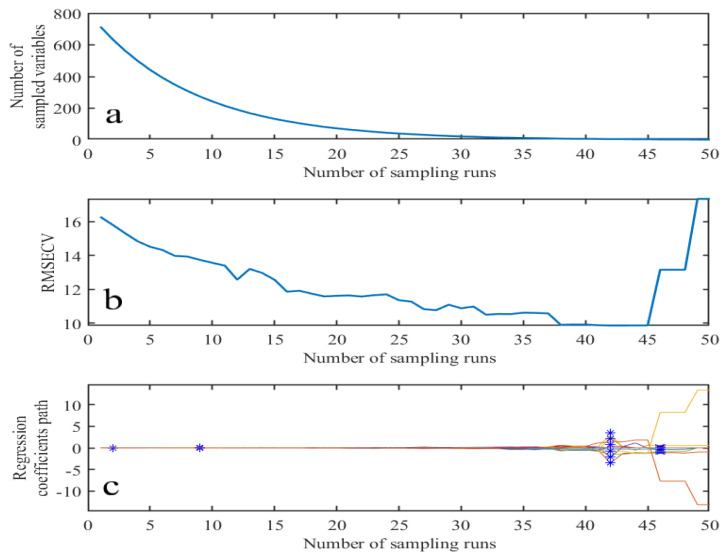
CARS photoacoustic spectroscopy feature wavelength extraction. (**a**) Number of sampled variables (**b**) RMSECV (**c**) Regression coefficient path. * is selected wavenumber variables.

To avoid the inaccurate extraction of characteristic wavenumber bands due to incomplete samples, this paper also used the CARS method combined with Monte Carlo sampling and the regression coefficient of the PLS model to extract the characteristic wavelengths. As shown in Figure 9, in the sampling process from 1 to 44, the number of wavenumber bands entering the next PLS model is gradually reduced due to the use of the exponential decay function to forcefully eliminate the wavenumber bands with small weights, resulting in a continuous decrease in the root mean square error of the cross-validation RMSECV value. After the 44th sampling, the RMSECV value shows an upward trend and a large value, so the number of characteristic wavenumber bands at the 44th sampling is selected. They characteristic wavenumber bands are1137, 1253, 1351, 1429, 1469, 1658, 1727, 1744, 1801 and 2904 cm^−1^.

#### 3.3.3. SPA-Based Photoacoustic Feature Extraction

**Figure 10:**
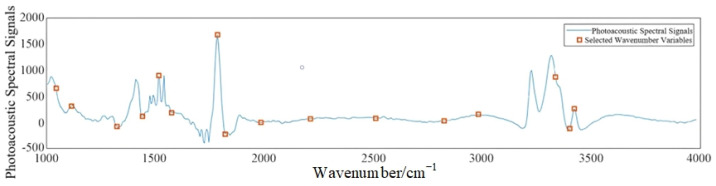
Photoacoustic spectral feature wave number extraction.

The principle of SPA variable selection is that the newly selected variable is one of all the remaining variables that have the largest projection on the orthogonal subspace of the previously selected variable. This method has the advantage of extracting several characteristic wavenumber bands of the full band region, and it can eliminate redundant information from the original data matrix. As shown in Figure 10, the wavenumbers of the eigenwaves selected by SPA are 1047, 1119, 1326, 1434, 1518, 1573, 1783, 1816, 1998, 2212, 2506, 2831, 2997, 3354, 3398, and 3424 cm^−1^.

### 3.4. Model Building and Validation

#### 3.4.1. Classification Results Based on the Weak Classifier

To obtain the best weak classifier, five weak classification models, namely, linear discriminant analysis, KNN, random forest, recommendation algorithm, and backpropagation, were established to model and analyze the photoacoustic spectral data of soybean oil and to combine the 5-fold cross-validation for the meta-model with the daily forward chain cross-validation. Training and tuning optimization were carried out. The training and test sets are shown in Table 2, using the optimal 500 Hz wavelength as the feature wavelength input.

From Table 2, it can be seen that the accuracy of the PCA method is low because this method has the disadvantage of extracting features with large wavenumber outlier points and the influence of negative factors in the original data, which will lead to the unclear meaning of the principal component evaluation function. Therefore, although the PCA method can extract relatively few principal components, it can obtain more than a 0.95 cumulative contribution rate, but, as a classification model, its accuracy is poor. The accuracy of the CARS-BPNN, SPA-KNN, SPA-PNN, and SPA-BPNN models is greater than 90%. Among them, PNN has the highest accuracy of nearly 95%, which is the best among the five weak classifiers.

The KNN, PNN, and BPNN classifiers were selected for stacking integration learning because it was necessary to keep the classification results of each classifier close and good when selecting weak classifiers for the stacking algorithm.

#### 3.4.2. Classification Results Based on Stacking Integration Learning

From the above study, it can be seen that, at a feature frequency of 500 Hz, 3 classifiers with close and optimal classification results, KNN, PNN, and BPNN, were selected as the first layer classifiers, and the PNN classifier, with the best modeling effect, was selected as the second layer classifier to build the stacking integrated learning classification model. From Table 3, it can be seen that the classification effect of soybean oil with different frying durations can be improved from 94.99% to 98.46% after training by the stacking integrated learning model.

## 4. Conclusions

Based on the above experiments and analysis, the self-developed PAS method can classify the frying time of soybean oil quickly and without damage, and with high precision and low cost. Biochemical titration analysis and comparison with the GB7102.1-2003 standard showed that the free fatty acid content of soybean oil after 48 h of frying exceeds 0.6%, and the acid value content exceeds 1.3 mg/g, which exceeds the maximum value allowed by the standard. Biological analysis showed that there were three obvious absorption peaks at 1747 cm^−1^, 2858 cm^−1^, and 2927 cm^−1^ for soluble sugars (main elements: C, H, O) and unsaturated oils (main elements: C, H, O). Combined with the PCA, CARS, and SPA algorithms, the influence of noise can be largely eliminated. In addition, the classification accuracy of the SPA-PNN-stacking combined model obtained by the integrated learning of stacking was 98.46%. The stacking integrated learning algorithm can effectively improve the detection accuracy of traditional classifiers. In this paper, the detection based on PCA is not sufficient, but a new method for edible oil quality detection is proposed.

## Figures and Tables

**Table 1:** Free fatty acid content and an acid value of soybean oil at different frying durations.

Frying Durations	Acid Price (mg/g)	Free Fatty Acids (%)
0 h	0.15	0.08
8 h	0.41	0.20
16 h	0.48	0.24
24 h	0.63	0.32
32 h	0.93	0.47
40 h	1.04	0.52
48 h	1.35	0.68
56 h	1.74	0.87
64 h	2.00	1.01
72 h	2.30	1.16
80 h	2.55	1.28

**Table 2:** Classification results based on weak classifiers.

Classifier	PCA		SPA		CARS	
	Training Set	Test Set	Training Set	Test Set	Training Set	Test Set
LDA	0.3733	0.3481	0.6519	0.7493	0.7547	0.8067
RF	0.4523	0.5739	0.9128	0.9428	0.8767	0.8407
KNN	0.5027	0.5198	0.7183	0.7903	0.8231	0.9384
PNN	0.6925	0.7003	0.6368	0.7602	0.8602	0.9499
BPNN	0.6163	0.6731	0.9317	0.9474	0.8909	0.9502

**Table 3:** Stacking integrated learning classification results.

Classifier	CARS		SPA	
	Training Set	Test Set	Training Set	Test Set
KNN	0.8401	0.8654	0.8967	0.9514
BPNN	0.9137	0.9582	0.9333	0.9454
PNN	0.8333	0.8705	0.9701	0.9846

## Data Availability

No new data are created.
